# Participatory mapping of target areas to enable operational larval source management to suppress malaria vector mosquitoes in Dar es Salaam, Tanzania

**DOI:** 10.1186/1476-072X-6-37

**Published:** 2007-09-04

**Authors:** Stefan Dongus, Dickson Nyika, Khadija Kannady, Deo Mtasiwa, Hassan Mshinda, Ulrike Fillinger, Axel W Drescher, Marcel Tanner, Marcia C Castro, Gerry F Killeen

**Affiliations:** 1 Department of Public Health and Epidemiology, Swiss Tropical Institute, Basel, Switzerland; 2 Department of Physical Geography, University of Freiburg, Freiburg, Germany; 3 Ifakara Health Research and Development Centre, Coordination Office, Dar es Salaam, United Republic of Tanzania; 4 City Medical Office of Health, Dar es Salaam City Council, Dar es Salaam, United Republic of Tanzania; 5 Ministry of Agriculture and Food Security, Dar es Salaam, United Republic of Tanzania; 6 Institute of Ecosystems Science, School of Biological and Biomedical Sciences, Durham University, Durham, UK; 7 Department of Population and International Health, Harvard School of Public Health, Boston, USA

## Abstract

**Background:**

Half of the population of Africa will soon live in towns and cities where it can be protected from malaria by controlling aquatic stages of mosquitoes. Rigorous but affordable and scaleable methods for mapping and managing mosquito habitats are required to enable effective larval control in urban Africa.

**Methods:**

A simple community-based mapping procedure that requires no electronic devices in the field was developed to facilitate routine larval surveillance in Dar es Salaam, Tanzania. The mapping procedure included (1) community-based development of sketch maps and (2) verification of sketch maps through technical teams using laminated aerial photographs in the field which were later digitized and analysed using Geographical Information Systems (GIS).

**Results:**

Three urban wards of Dar es Salaam were comprehensively mapped, covering an area of 16.8 km^2^. Over thirty percent of this area were not included in preliminary community-based sketch mapping, mostly because they were areas that do not appear on local government residential lists. The use of aerial photographs and basic GIS allowed rapid identification and inclusion of these key areas, as well as more equal distribution of the workload of malaria control field staff.

**Conclusion:**

The procedure developed enables complete coverage of targeted areas with larval control through comprehensive spatial coverage with community-derived sketch maps. The procedure is practical, affordable, and requires minimal technical skills. This approach can be readily integrated into malaria vector control programmes, scaled up to towns and cities all over Tanzania and adapted to urban settings elsewhere in Africa.

## Background

### Urban malaria and its historical control in Dar es Salaam

Malaria is responsible for more than one million deaths world-wide each year, mainly in sub-Saharan Africa [1-3]. In areas prone to malaria, urbanization has major implications for the transmission and epidemiology of malaria [4,5]. Although malaria vector density is typically much lower in urban areas compared to periurban and particularly rural areas [6], malaria transmission in urban and periurban settings remains a significant problem [2,3,7-9]. In Dar es Salaam, over a million malaria cases are reported by the health facilities every year [10], though malaria is clearly grossly overreported [11-13] and a considerable part of the infections might result from travel to rural areas [13]. Ninety percent of all malaria cases in Dar es Salaam are caused by *Plasmodium falciparum*, and the main vectors in this major urban centre are *Anopheles gambiae sensu strictu *Giles, *Anopheles arabiensis *Patton, *Anopheles funestus *Giles and *Anopheles merus *Dönitz [14].

Dar es Salaam has a long history in malaria control, starting more than 100 years ago in the German colonial era [15,16]. A variety of techniques has been used to control malaria in the city for many decades, with considerable success [14]. In 1972, economic and political reasons led to chemotherapy being the only anti-malaria intervention left in place. As a result, the density of *Anopheles *mosquitoes started to increase again [17]. Starting in 1988, the City of Dar es Salaam collaborated with the Japan International Cooperation Agency (JICA), focussing on vector control, promoting people's perceptions of malaria and involving communities in environmental management activities [14,16]. Despite some successes such as the rehabilitation of drainage infrastructure, this programme did not achieve sustainability and ended in 1996. Main reasons for this are considered to be insufficiently developed local ownership and capacity [16].

### The Dar es Salaam Urban Malaria Control Programme

**Figure 1:**
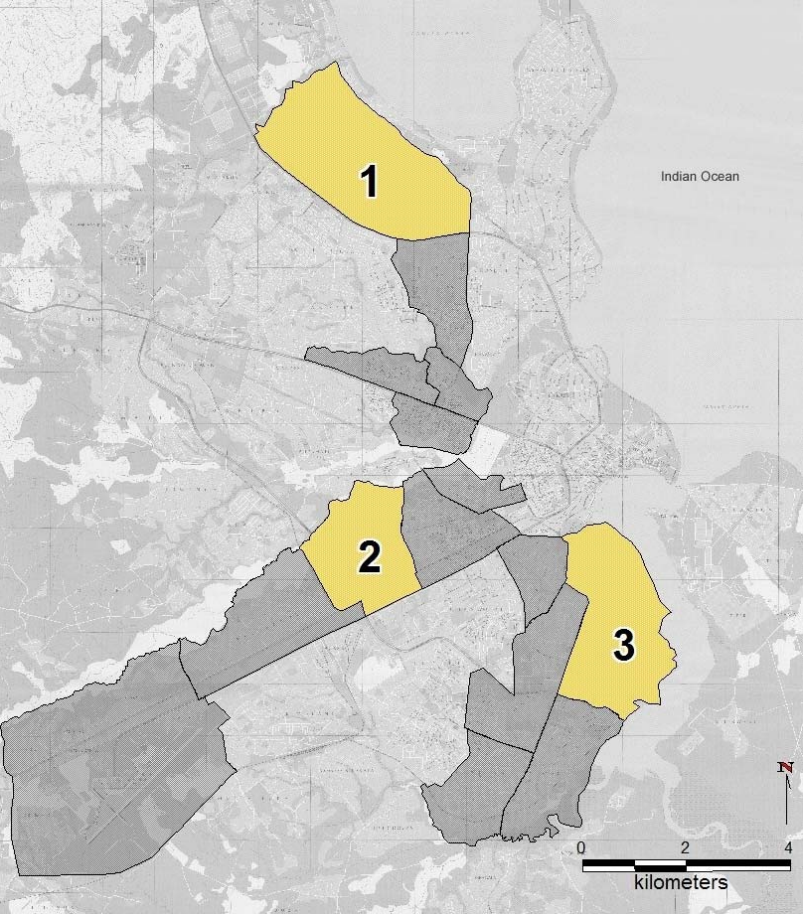
**Project wards of the Dar es Salaam Urban Malaria Control Programme**. Dar es Salaam City Map. The dark grey and yellow shaded areas show the 15 wards in which the UMCP is operating at present. The yellow areas mark the first three wards that were systematically mapped using the methodology described here. 1 – Mikocheni, 2 – Buguruni, 3 – Kurasini. Source of background map: Dar es Salaam City Map and Guide, published in 1995 by Surveys and Mapping Division, Tanzania.

**Figure 2:**
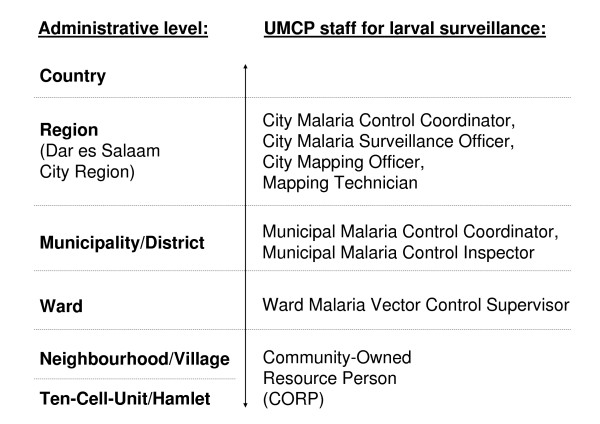
**Administrative levels in Tanzania and UMCP larval surveillance**. For purposes of clarity, the above overview only encompasses staff working full or part time for and paid by the UMCP. In fact, there are more institutions involved in programme implementation on each administrative level [42], such as the city medical officer, the municipal health board, the ward executive officer and the neighbourhood health committee.

The aim of the current Dar es Salaam Urban Malaria Control Programme (UMCP) is to control aquatic-stage mosquitoes using community-based resource persons (CORPs), and to evaluate the effectiveness of this intervention (for definitions and abbreviations please refer to table 1). The goals of the UMCP are part of the National Malaria Control Programme, which comprises vector control, improved malaria case management, malaria prevention in pregnancy, epidemic management and strengthening of systems for delivering and managing these interventions [18]. The UMCP has developed out of a local initiative: in 2002, the Ilala Municipal Council independently planned, funded and implemented a community-based mosquito surveillance programme. Two years later, this programme was scaled up to 15 of the 73 wards of Dar es Salaam, covering an area of 56 km^2 ^(figure 1), with support from national and international academic partners [16]. All UMCP activities are coordinated by the City Medical Office of Health, and are fully integrated into the decentralized administrative system in Dar es Salaam (figure 2). The UMCP in its current form was launched in March 2004 and operates on all five administrative levels of the city: the city council, 3 municipalities, 15 wards, 67 neighbourhoods referred to as *mitaa *in Kiswahili (singular *mtaa*, meaning literally street) and more than 3000 ten-cell-units (table 1 & figure 2). The main tasks on the four upper levels are project management and supervision, whereas the actual monitoring, mosquito larval surveillance and control is organised and implemented at the level of the smallest administrative units, the so-called ten-cell-units (TCUs). A TCU typically comprises about ten houses, in some cases even more than one hundred. Each TCU is headed by an elected chairperson [16,19]. The core activities of the UMCP are implemented by CORPs to whom specific responsibilities and target areas are allocated (table 1 & figure 2). On a weekly basis, they monitor and document the larval habitats of mosquitoes in every ten-cell-unit, receiving minimal remuneration [16,20]. The exact number of ten-cell-units that each CORP is responsible for varies depending on their sizes and characteristics. Starting in March 2006, additional CORPs have been recruited and trained to apply biological larvicide (*Bacillus thuringiensis *var. *israelensis*) to all potential larval habitats of malaria vectors. Thus, there are two groups of CORPs: the larval surveillance CORPs and the larviciding CORPs. This article only refers to those CORPs who are responsible for larval surveillance and monitoring.

**Table 1:** Definitions and abbreviations

**CORP:**	Community-Owned Resource Person. The responsibility for routine mosquito surveillance in Dar es Salaam is delegated to individual community members (CORPs) who are appointed and managed through neighbourhood health committees [16].
**GIS:**	Geographical Information System. A GIS is a computer system for capturing, storing, analyzing and managing data and associated attributes which are spatially referenced to the earth [32, 33].
**GPS:**	Global Positioning System [32, 33].
**Municipality:**	The Dar es Salaam City Region is subdivided into three municipalities, namely Ilala, Temeke and Kinondoni.
**Open Space:**	Public or private unbuilt land, for example hazardous lands declared not suitable for construction, road reserves, available land for community use, as well as residential, industrial or institutional plots under-utilised or awaiting development. Open spaces are often used for agricultural activities.
**Plot:**	All TCUs within the wards covered by the UMCP are subdivided into plots. A plot is defined here as a specific physical area with an identifiable owner, occupant or user and with clearly defined boundaries within one specific TCU. The plot boundaries are defined by UMCP staff. Therefore, the plots do not always correspond with actual cadastral information such as land ownership [31].
**Neighbourhood:**	The 73 wards of the Dar es Salaam City Region are administratively subdivided into 368 neighbourhoods. The 15 wards covered by UMCP comprise 67 neighbourhoods. The local Kiswahili term for neighbourhood is *mtaa *(plural *mitaa*) which literally means "street".
**TCU:**	Ten-Cell-Unit. The 368 neighbourhoods (*mitaa*) of the Dar es Salaam City Region are subdivided into several thousand ten-cell-units (TCUs). They typically comprise about ten houses, in some cases even more than one hundred, and are headed by an elected chairperson. Some non-residential areas were not included in any TCU by the time the UMCP started. In the progress, such areas were assigned to be new TCUs, or were attached to existing TCUs in all neighbourhoods within the UMCP.
**UMCP:**	Urban Malaria Control Programme of the Dar es Salaam City Medical Office of Health, in co-operation with national and international research and funding organizations.
**Ward:**	The three municipalities of the Dar es Salaam City Region are subdivided into 73 wards. Currently, 15 of these wards are covered by the UMCP.

Targeting of the most productive habitats could improve the cost-effectiveness of mosquito larval control in Africa [21]. Remotely sensed imagery, GIS and GPS can support the identification and recording of such habitats on village or city level [14,22-26]. However, the operational challenges of a large-scale programme and the lack of scientific evidence of its feasibility and effectiveness suggest the need for exhaustive coverage and very simple implementation protocols that can be implemented by community-level staff with minimal education. This is essential if larval control is to achieve substantial reductions of malaria transmission and disease burden [27], considering the highly dynamic nature of mosquito larval habitats in urban areas that are often too small to be identifiable on aerial pictures [25,27]. In order to provide the necessary basis for this goal, the CORPs record and monitor all potential mosquito habitats. However, an evaluation of the CORPs work in the first months of the programme from March to June 2004 revealed the need for new procedures such as the ones presented in this paper, in order to tighten up the standards of community-based larval control. During the previous evaluation, the CORPs detected less than 50% of all breeding sites [20], which is unlikely to achieve satisfactory impact on malaria transmission if this is considered an upper limit for coverage with insecticide [27].

This article is part of a series of papers that present results from different elements of the UMCP programme [14,16,20,25]. Future articles will show that larval control does work and is effective in reducing malaria burden in Dar es Salaam (unpublished data), and specify the surveillance methodology. This paper describes a community-based participatory mapping procedure that can be used to overcome the challenges described above, and allows the integration of the valuable knowledge of community members. The approach aims to enable complete coverage of targeted communities with larval control by optimising the quality and spatial coverage of community-derived sketch maps. The overall goal of developing this procedure is to improve programme management systems in a way that makes routine larval surveillance and control truly effective. The approach described is easily replicable, adaptable and transferable to any other city in Tanzania or Africa, provided the necessary resources and policy support are available. It particularly takes into account the resource situation and limited availability of maps and remote sensing data in such settings, which cannot be compared to western countries.

## Methods

### Study area

This study was conducted in urban Dar es Salaam, the largest city and *de facto *capital of Tanzania with almost 2.7 million inhabitants in 2005 [28]. Situated on the shores of the Indian Ocean, with large parts of the city located only a few meters above sea level, Dar es Salaam has a hot and humid tropical climate with two rainy seasons and is characterized as an area with endemic and perennial malaria, with transmission occurring during the entire year [29]. The administrative city region covers an area of almost 1,400 km^2 ^[14], of which 56 km^2 ^are covered by the fifteen wards included in the UMCP at present (figure 1). Although the UMCP area only makes up for 4% of the overall city region, it covers some of the most densely populated parts of the city. It is inhabited by more than 610,000 people, and therefore almost a quarter of the total population [30]. Most of this area is built-up, but nevertheless provides excellent breeding sites for mosquitoes [14,25], especially where the groundwater table is high. In Dar es Salaam, almost all kinds of water accumulations can be breeding sites for *Anopheles sp*. larvae [25]. The participatory mapping procedure described here was developed in three of the fifteen UMCP wards. These three wards had been selected as study area because the UMCP had chosen them as the first wards to implement community-based larval control starting from 2006.

### Resources needed

The resources and materials necessary for the creation of sketch maps and their verification are listed in table 2, together with an indication of their respective costs.

**Table 2:** Costs of participatory mapping

**Activity/Item**	**Costs in TSh (2005)**	**Costs in USD**	**Costs per km^2 ^in USD**	**Costs per TCU in USD**
Technician (BSc in Geography or equivalent; skilled in working with communities, GIS and aerial imagery)	2,450,000	2,156	129	3.7
Field assistant (trained by technician)	1,050,000	924	55	1.6
CORPs (responsible for and familiar with the area to be mapped, and respected by the community)	780,000	686	41	1.2
Training for CORPs in sketch mapping (implemented by UMCP management team and technician)	200,000	176	10	0.3
Aerial photographs (printed; resolution of 1 m or better and colour images recommended)	5,000,000	4,400	262	7.5
Lamination (for protection of printed aerial photographs)	50,000	44	3	0.1
Stationary & copy costs (mapping templates for TCU sketch maps, description forms for each plot, pens)	300,000	264	16	0.4
Computer with GIS software (for example MapInfo Professional 6.0, ArcView 3.2 or more recent versions)	2,500,000	2,200	131	3.7
Colour printer & cartridges	1,200,000	1,056	63	1.8
Motorbike (optional) & fuel	2,300,000	2,024	121	3.4

**Total**	**15,830,000**	**13,930**	**831**	**24**

The costs of the participatory mapping for a total of three wards (16.8 km^2^) with a total of 589 TCUs are calculated on the following basis: Two CORPs per day with a daily remuneration of TSh 3,000 per person, with five working days per week over a period of six months. Exchange rate: TSh 1,000 = USD 0.88 (source: , September 1, 2005). The amount for item "aerial photographs" may vary due to availability; adequate imagery might be freely available, e.g. from Google Earth™.

### Preliminary sketch mapping

**Figure 3:**
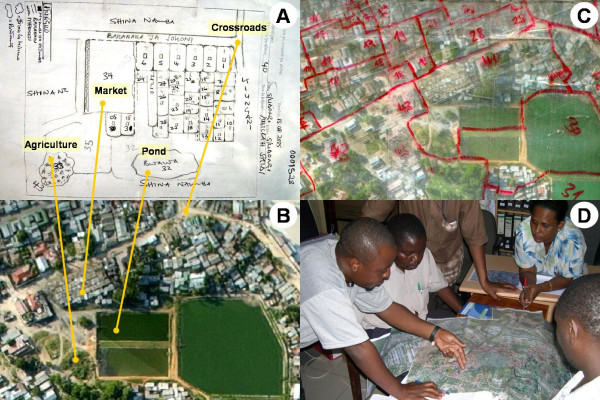
**Example of a sketch map, aerial picture and technical map**. A. Sketch map of TCU 40 in Kurasini ward, Shimo la Udongo neighbourhood, as drawn by the responsible CORP. Features comprise plots with continuous numbering, streets, drains, agricultural areas and ponds. B. The same area on the aerial picture. The yellow lines are connecting identical features on the sketch maps and the aerial picture. C. The same area on the laminated aerial photograph used for the technical mapping in the field. The features to be mapped were marked with non-permanent marker pens. Red: TCU boundaries and TCU numbers. D. Project management team discussing over the technical map of a whole ward, and deciding on necessary follow-up actions.

The framework within which the daily work of the CORPs is conducted is a set of sketch maps of their areas of responsibility (figure 3A). Their creation is the first activity in the mapping sequence described here. The first round of sketch mapping in Dar es Salaam was initiated in 2004. The CORPs drew preliminary sketch maps of all existing TCUs located within the programme area. For this endeavour, they were supported and trained by UMCP staff, and provided with printed standard operating procedures as reference material [31] (additional file 1; Guidelines for ten-cell-unit mapping). The original sketch maps and corresponding description forms were filed at the respective ward offices, and a copy brought to the city council for central information management.

The purpose of the sketch maps is to enable the CORPs to assign a unique number to any larval habitat found within a plot and enable supervisory staff to identify it unambiguously when inspecting the work of that CORP. Features included in the sketch maps are roads, pathways, drains or other landmarks, boundaries of the TCUs, and a subdivision of the whole TCU area into individually numbered plots based on regular use or ownership [31]. Attached to every sketch map is a description form that contains details about each plot such as house number, name of the household head or characteristics of the area [31] (table 1 and additional file 1). There is one sketch map for each TCU.

The sketch maps do not necessarily look like the area itself from the air (figure 3B), but nevertheless provide good guidance for the CORPs in the field. One obvious advantage of the system is that it corresponds to the existing administrative boundaries. This makes it easier for the CORPs to orient themselves in the field, as most community members are aware of the number of the TCU their household is located in, and thus can be asked if in doubt.

### Technical mapping with aerial photographs

In the next step, which will be referred to as "technical mapping" as opposed to "sketch mapping", the preliminary sketch maps were verified, corrected and formalized in the field by a technical team in collaboration with the CORPs. By using aerial photographs, all boundaries of TCUs, neighbourhoods and wards were formally mapped. The basis for the technical mapping was a digital aerial picture of Dar es Salaam in colour, taken in 2002 (ground resolution 0.5 m, produced by Geospace International, Pretoria, South Africa). This picture covers the whole urban area of Dar es Salaam. The relevant segments of the picture were colour printed as a mosaic of A4 pages at a scale of 1:3,000. The prints were laminated in order to protect them during intensive use in the field, and to allow drawing on the transparent surface with non-permanent marker pens that can easily be erased again for corrections (figure 3C). Finished parts of the map were covered with transparent sticky tape for protection of the drawings.

**Figure 4:**
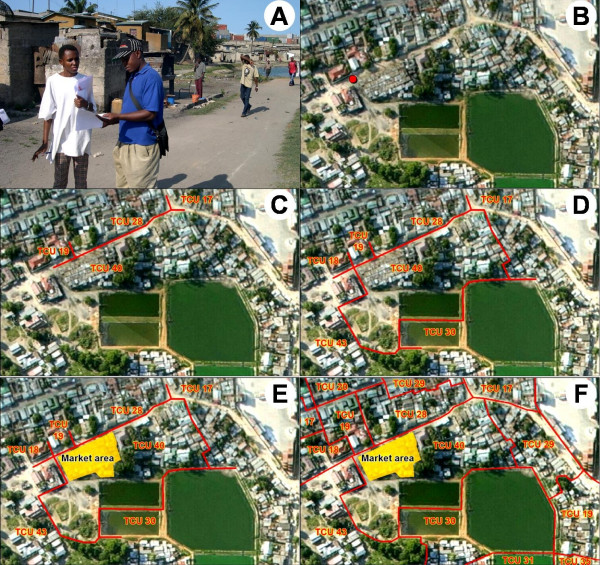
**Technical mapping of a TCU**. The underlying scene is taken from the aerial picture and shows TCU 40 and its surroundings in Kurasini ward, Shimo la Udongo neighbourhood. The large green structures in the lower half of the picture are sewage ponds. A. Mapping technician and CORP walking along the boundary of the TCU. The sewage ponds are visible in the background. B. The starting point is marked on the map. C. Marking the boundary of TCU 40 in red colour while walking along the boundary. The neighbouring TCU numbers and three-way-intersections are marked at the same time. D. Reaching the starting point again. E. The technical mapping of TCU 40 is finished. The market area has been identified as unsurveyed and is marked in yellow colour. F. Final technical map including the neighbouring TCUs.

After meeting all stakeholders, including the CORPs, at the local government office, the area to be technically mapped on the respective day was agreed on. The technical team showed a sample map so that everybody could understand how the technical map should appear in the end. This particularly helped avoiding the potential misunderstanding on the side of the CORPs that the technical team came to evaluate their work with the possibility of disciplinary action. Such perceptions proved very counterproductive because they greatly limited open interaction between the CORPs and the technical mapping team. The technical team and the responsible CORPs then went to the nearest TCU he or she was working in, together with the respective preliminary sketch maps (figure 3A), description forms, and laminated aerial photograph (figure 3C &3D). The CORP was asked to take the team to the boundary of the TCU. After reaching it, the position was marked on the photograph as the starting point (figure 4B). The team then walked along the boundary with the neighbouring TCU. The boundary was continuously marked on the photograph (figure 4C), and regular stops were made to verify accuracy. While walking, the CORPs were asked repeatedly which TCU was on the left side, and which one on the right. As soon as another border with a different adjoining TCU was reached, the team marked the three-way intersection of the TCU being mapped and the two adjacent TCUs (figure 4C &4D). This procedure was continued until the starting point was reached again (figure 4D). If it was not possible to walk along the boundary due to construction or other obstacles, it was ensured that what was marked in the technical map represented the actual agreed border. With the same procedure, all existing TCUs within a ward were mapped. By doing so, previously unsurveyed areas were identified and included into the sketch maps.

### Identification of missing areas and correction of sketch maps

Some of the identified unsurveyed areas were relatively small and easy to integrate into the sketch maps, whereas others turned out to be very large and required a more complex follow-up action by inclusion into newly created TCUs. Problems related to small areas could be solved directly on the spot. After the technical mapping of each single TCU, the team thoroughly checked for unsurveyed areas within that TCU. The sketch map had to cover exactly the same area as marked on the aerial photograph, and all areas within the TCU had to be assigned to a specific plot so that all plots could subsequently be surveyed by CORPs for mosquito larval habitats on a regular and routine basis. Omissions of certain areas from the sketch maps were immediately corrected by assigning a new plot number or by adding an area to an existing plot on the sketch map. Any unsurveyed areas included by the technical team were marked for documentation, and included in the sketch maps and description forms immediately. This means that the TCUs defined by the UMCP are not always identical to administrative TCUs in terms of their boundaries.

In the case of access-restricted, relatively large unsurveyed areas that did not belong to any TCU, new TCUs were created by the CORPs together with their supervising staff. The boundary lines of the new TCUs were defined, and new TCU numbers assigned. Permission for regular access to all properties located within the new TCUs was sought and obtained by the programme management on municipal level. Finally, the new sketch maps were formalized and corrected in exactly the same way as described above.

### Digitization of technical maps and provision for operational teams

As the last working step, the technical maps based on the aerial imagery were digitized. Provided that the aerial imagery used is available in digital format, only a computer with GIS software is needed. Digitization and data analysis was done with the GIS software package MapInfo Professional^® ^7.0 [MapInfo Corporation, One Global View, Troy, New York 12180]. The aerial imagery was georeferenced, which means that geographical coordinates (UTM, longitude/latitude) were available for each point of the image. This is the case with most commercial remotely sensed imagery, but can also be done by identifying a few reference points of which the coordinates are known (possible sources are topographical maps or a standard GPS receiver) [32,33]. The digitizing itself was done "on screen" with a computer, by creating separate layers for TCUs, neighbourhoods, wards, unsurveyed areas and text labels. The latter comprise useful features such as landmarks and street names, and consist of points with attribute data. All other layers consist of polygons with attribute data such as TCU numbers, names of wards, neighbourhoods and names of responsible CORPs, characteristics of each area and automatically calculated sizes of each polygon.

**Figure 5:**
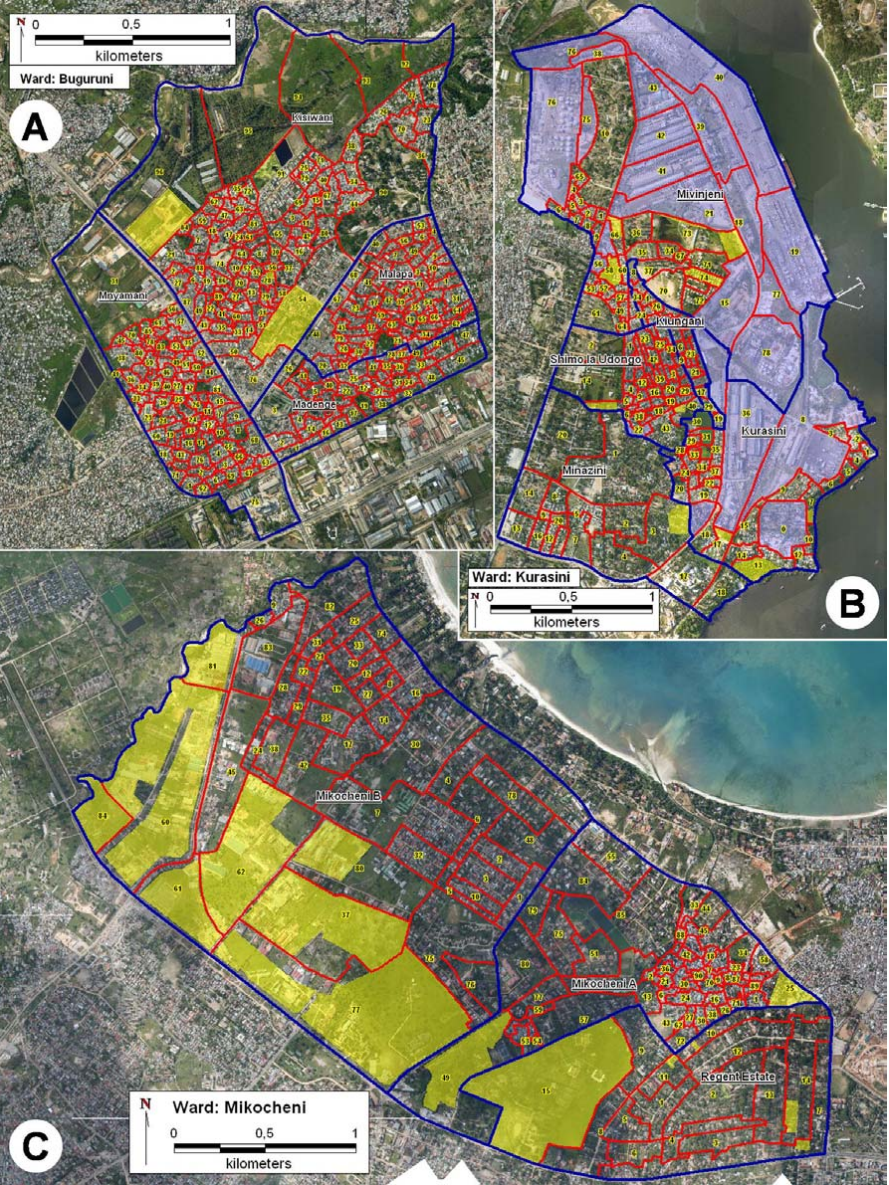
**Final technical maps of the three study wards**. A. Buguruni ward. B. Kurasini ward. C. Mikocheni ward. Red lines: TCU boundaries, numbers: TCU numbers, blue lines: neighbourhood boundaries, yellow areas: initially unsurveyed by CORPs, blue areas: initially unsurveyed by CORPs and now included in newly created TCUs.

After digitization, each ward and the mapped features were printed as colour maps (figure 5). One colour map per ward was kept on file at the city office together with copies of all corrected sketch maps and description forms. A large-scale colour print of each ward map was laminated and returned to the respective local government offices, where the originals of the sketch maps and description forms are stored while not in use by the CORPs. During operations, the colour maps are mostly used by supervisory staff for evaluation of the CORPs work and assurance of complete larval control coverage.

### Ethical considerations

All work during this study was on geographical material and did not involve human subjects. Research clearance was obtained from the Medical Research Coordination Committee of the National Institute of Medical Research in Tanzania (NIMR/HQ/R.8a/Vol. IX/279) and the Tanzanian Commission of Science and Technology (No. 2004-69-MFS-2004-24 and No. 2005-123-NA-2004-163). This manuscript has been published with kind permission of the Director of the National Institute for Medical Research of the United Republic of Tanzania. In order to achieve community consent and before starting any field work, the stakeholders and community leaders at the respective local government units were contacted. The goals of the activity were explained, and the mapping team was introduced. All responsible CORPs and the programme management staff in charge for an area to be mapped were present in such meetings.

## Results

**Figure 6:**
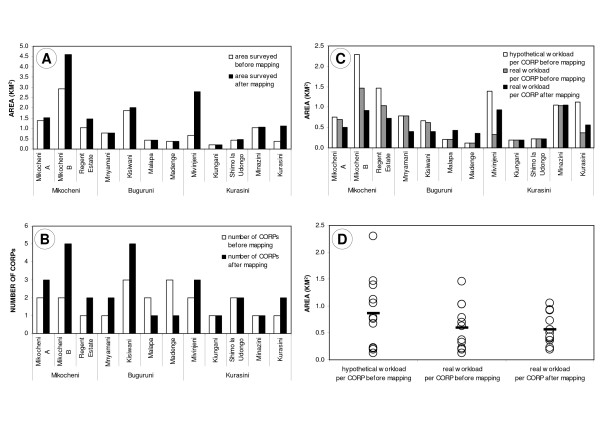
**Impact of technical mapping**. Situation in each of the 12 neighbourhoods of the study area, directly before and three months after the preliminary sketch maps were formalized and corrected by using the technical mapping approach with aerial photographs. A. Total area in km^2 ^surveyed by CORPs (*) before and after technical mapping. The latter is equal to 100% of the surface area of all 12 neighbourhoods. B. Number of CORPs before and after technical mapping. C and D. Average weekly workload per CORP in km^2 ^(**) before and after technical mapping. "Hypothetical workload" assumes that the sketch maps had been correct and leaving no gaps within each neighbourhood from the beginning, and that the CORPs had surveyed all these areas even before the sketch maps were verified. "Real workload" only takes into account the area actually surveyed by CORPs. The circles represent the 12 neighbourhoods, and the horizontal bars represent the arithmetical means. (*) area which is routinely and weekly monitored by CORPs, and for which sketch maps plus description forms are available (**) weekly workload is calculated as the total area of a neighbourhood divided by the number of CORPs working in the respective neighbourhood

Three complete wards covering a total area of 16.8 km^2^, consisting of 12 neighbourhoods and 589 TCUs, were mapped during several phases with interruptions between March 2004 and January 2006 (figure 5). The mapping comprised the community-based creation of sketch maps and their verification by a technical team using laminated aerial photographs. The mapped area is equivalent to 30% of the total project area and home to 128,000 people [30]. The total time needed for the actual work was six months. Overall, it was found that before the technical mapping, only 14.0 km^2 ^(83.3%) of the study area had been included in TCUs, and only 11.5 km^2 ^(68.4%) of the study area had been surveyed for mosquito larval habitats by CORPs (figure 6A). This means that by that time, 2.8 km^2 ^(16.7%) of the survey area were not covered by existing TCUs or any sketch maps. Even where TCUs existed and sketch maps for those were available, 2.5 km^2 ^(14.9%) of those TCUs were not represented in the sketch maps or surveyed by CORPs. Immediately after their identification, all these shortcomings were solved by either adding areas to existing sketch maps (figure 5, areas marked in yellow), or by creating new TCUs and corresponding sketch maps where necessary (figure 5, areas marked in blue). For the purposes of facilitating surveillance and management activities, additional TCUs were created as a result of division of larger TCUs. Overall, the total number of TCUs grew by 27 (4.8%) compared to the first round of sketch mapping.

In the course of the technical mapping, shortcomings of the TCU-based surveillance system were identified and eliminated. All of them initially contributed to gaps in terms of areas that were not surveyed for mosquito larval habitats by any CORP. Non-residential areas such as industrial areas, commercial areas and open spaces (table 1) are not usually part of any TCU or residential lists. Therefore, they often were not included in preliminary sketch maps. Furthermore, some CORPs at first did not understand that such areas are important for their work, and tended to focus on residential areas. Other initially unsurveyed areas resulted from misinterpretation of actual TCU boundaries by the CORPs. Such misinterpretations often happened where the boundaries between TCUs did not coincide with intuitive landmarks such as roads, but were located in less structured areas such as river valleys without residential areas. In such cases, all responsible CORPs including those from adjacent TCUs, their supervisors and the technical team revisited the area. The borders between their respective areas could then be assessed properly with full participation by all responsible for and familiar with the area.

### Mikocheni ward

Mikocheni (figure 5C) with a total area of 7.6 km^2 ^is subdivided into three neighbourhoods (Mikocheni A, Mikocheni B and Regent Estate). The northern parts of Mikocheni A and B as well as the whole of Regent Estate are low and medium density residential areas, with residents of relatively high socio-economic status. Plots in this area are typically well defined by walls or fences. The same is true for the industrial and commercial areas in the south of Mikocheni B. For the CORPs this means that they have to ask watchmen or owners for permission every time they want to enter such plots, which was the main reason for not surveying such properties for larval habitats initially. In Regent Estate, there were some residential plots where the CORPs were not allowed to enter, or were not willing to do so because of watchdogs or the intimidating reputation of the owner. All these cases were effectively resolved by informing the municipal malaria coordinator, who ensured access by writing formal letters and arranging meetings with the concerned companies and individuals. A special arrangement was necessary for TCU 84 at the western corner of Mikocheni B, which is owned by the armed forces. In this case, the solution agreed between army representatives and the municipal malaria coordinator was to recruit an extra CORP from among the army personnel. Notably, no problems occurred in the cluster of unplanned residential TCUs in the east of Mikocheni A. In this area, characterized by high housing density and low socio-economic status, only few plots are fenced, and access was granted in all cases. The initially unsurveyed area in the eastern corner of Mikocheni A belongs to a shopping mall. The planned and unplanned areas can be easily distinguished in the colour maps by the different courses of their TCU boundaries. In planned areas, the lines are relatively straight and smooth, but they appear rather irregular and uneven in the unplanned part in the east of Mikocheni A. In addition, TCUs in planned settings are generally a lot larger compared to those in unplanned ones, due to the size of the houses and corresponding plots.

### Buguruni ward

**Figure 7:**
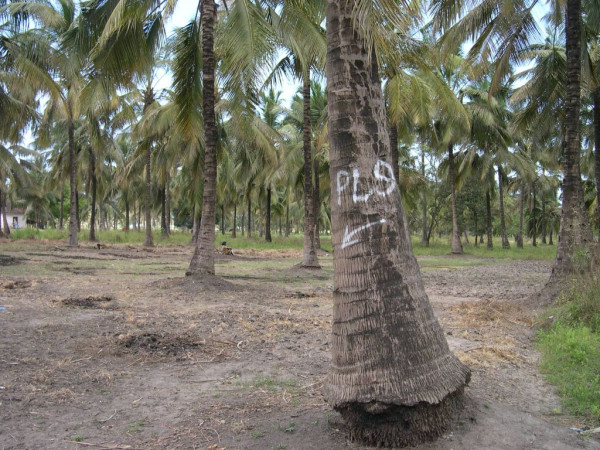
**Painted coconut tree**. For better orientation, the responsible field & supervisory staff painted plot and TCU numbers on coconut trees in a large uninhabited agricultural open space of Buguruni ward (TCU 92–96).

Buguruni ward (figure 5A) with a total area of 3.6 km^2 ^consists of four neighbourhoods (Mnyamani, Kisiwani, Malapa and Madenge). Although it is a lot smaller than Mikocheni, it consists of more than twice as many TCUs. This reflects the fact that the residential parts of Buguruni are largely unplanned settlements with high housing density and a relatively low socio-economic background, characterized by small TCU sizes. The few relatively large TCUs are industrial areas, belong to the police and churches, or are used for agricultural purposes such as the northern part of Kisiwani which is located in a river valley (TCU 92–96). In order to ease the work for the CORPs in this huge agricultural area, the local field and supervisory staff came up with a special way for defining TCU and plot boundaries. These initiatives were initiated by the technical mapping, during which confusions about TCU and plot boundaries on behalf of the CORPs became obvious. The collaboration with staff on all administrative levels stimulated creative, participatory and solution-oriented action. Comprehensive larval surveillance was achieved by using coconut trees as boundary indicators, and by marking their stems with the respective numbers (figure 7). The few unsurveyed areas that had to be included were all due to the initial restriction of the owners to let the CORPs enter their plots.

### Kurasini ward

Kurasini ward (figure 5B) comprises five neighbourhoods (Mivinjeni, Kiungani, Shimo la Udongo, Minazini and Kurasini). It is characterized by large commercial harbour areas and petrol industries located in the northern and eastern parts. All of these areas were initially not surveyed and had to be included into the surveillance system. Most of them were not part of existing TCUs. Therefore, 22 new TCUs and corresponding sketch maps were created by the responsible field and supervisory team (blue areas in figure 5B). Access to those areas was established through formal letters to and discussions with company representatives. The south-western half of Kurasini ward is residential area. Similar to the situation in Mikocheni, there are planned low and medium density as well as unplanned high density settlements, which can be distinguished by their differing TCU sizes and boundary characteristics. Within the residential areas, several initially unsurveyed areas were included into the survey system. Three areas were found unsurveyed due to their uninhabited status, namely a bush area, a mangrove swamp, and an open space. A commercial area was included in Minazini. The remaining initially unsurveyed areas had been difficult to access, such as fenced industrial plots. However, these areas were readily assimilated through intervention of senior municipal staff, as described for Mikocheni.

The technical mapping revealed that the sizes of TCUs vary tremendously. The smallest TCU (0.0013 km^2^) was found in Buguruni Malapa. Before the sketch maps were corrected, the largest TCU with almost 0.9 km^2 ^was located in Buguruni Kisiwani. After subdividing it into the smaller TCUs 92–96, the maximum TCU size today is 0.6 km^2 ^(TCU 77 in Mikocheni B). Apparently, these variations had not been adequately considered in the initial allocation of work areas to CORPs. Some CORPs had been assigned relatively small areas, whereas others were responsible for much larger areas (figure 6A and 6B). The technical mapping led to the redistribution and reallocation of the work areas per CORP, and the recruitment and training of additional CORPs where necessary (figure 6B and 6C). The average weekly workload per CORP is defined here as the surface area of a neighbourhood divided by the number of CORPs assigned to this neighbourhood. Hypothetically, i.e. if the sketch maps had been correct and leaving no gaps within each ward from the beginning, and if the CORPs had really surveyed all areas before the sketch maps were verified, this would have resulted in an average weekly workload of almost 0.9 km^2 ^per CORP (figure 6C and 6D). In reality, only 0.6 km^2 ^per CORP and week had been surveyed on average, which lead to large unsurveyed gaps equivalent to a 31.6% shortfall in spatial coverage. After the correction of the sketch maps, each CORP is now responsible for an average of slightly less than 0.6 km^2 ^per week. Considering the average weekly workload of all CORPs, the largest area to be surveyed by a CORP has decreased from 2.3 km^2 ^before the technical mapping to 1.1 km^2 ^afterwards. At the same time, no CORP was responsible for surveying less than 0.2 km^2 ^after the technical mapping, compared to 0.1 km^2 ^before (figure 6C and 6D). Thus, the workload per CORP has been distributed more equitably, which is likely to impact the quality of work.

## Discussion

The community-based participatory mapping represents a useful tool for urban mosquito larval control. After its completion, corrected sketch maps, description forms and formalized colour maps based on an aerial photograph were available for the complete study area. On this basis, 100% spatial coverage of mosquito larval habitat surveillance by CORPs was achieved, which would have been impossible with either the sketch maps or the formalized colour maps alone.

From the point of view of programme field workers including CORPs, the sketch maps and associated detailed plot descriptions are indispensable guidance tools. The sketch map system accommodates the different cognitive abilities of the CORPs, as the map style can be adapted according to their personal preferences in order to achieve optimal orientation. However, only few CORPs were comfortable to use an aerial photograph as a basis for their work, which rules out the option of replacing all sketch maps with formalized maps. Nevertheless, CORPs who wish to use formalized maps as an addition to their sketch maps can be provided with laminated printouts. When a CORP has to be replaced, the successor takes over the existing sketch maps but is free to adjust or redraw them if desired.

From a programme management perspective, the sketch maps are an ideal method to assign a unique number to each plot, whereas the technical mapping approach with aerial imagery proved to be essential for the verification and correction of the sketch maps. Moreover, the georeferenced colour maps that show the demarcations and locations of TCUs enable management staff to assess and analyze the data collected by the CORPs, and to conduct targeted spot checks.

The use of GIS software in the mapping approach proved to be extremely helpful for programme management and supervision of field activities, although only basic functions were utilized. Similar positive findings have also been made in other malaria control programmes in South Africa and Mozambique [34-36], and public health in Africa generally [37]. The approach does not require any electronic devices such as GPS receivers in the field. In addition, if digital aerial imagery is available, costly equipment like digitizing tablets or large format scanners are not needed. The entire GIS database as well as all subsequent updates thereof has been made available to the central GIS unit of the Dar es Salaam City Council. It can be used as a basis for any Council activity such as health interventions, waste management programs, and urban planning, to name a few.

The mapping approach adheres to the existing administrative boundary system in Tanzania, mainly referring to the ten-cell-units. In a dynamic environment such as the rapidly growing city of Dar es Salaam, this allows optimal orientation for community-based programme staff in the field, without having to create entirely new sets of artificial boundaries. Whenever there are changes, sketch maps can easily be updated during their weekly use by the CORPs. The technical team only needs to be informed in case TCU boundaries have been modified. It is argued that this approach has practical programmatic advantages over imposed raster grid systems [23,38,39], because it considers user-definable boundaries that can be agreed in a participatory manner on the ground and that can be readily recognized by community-based staff without access to, or the necessary education to use, GIS technology. In this way, GIS can be participatory, with the potential to enhance community involvement [40]. In the operational context of malaria control Dar es Salaam, this rather basic but straightforward way of applying GIS is advantageous, as resources in terms of available data and expert personnel are limited. The same tendency has also been observed for lower-income countries in general [41], and accessing such limited resources can be a challenge in itself.

The system of ten-cell-units such as the one in Dar es Salaam (or *hamlets *and *vitongoji*, as they are called in the rural districts of Tanzania [42]) probably is slightly different to the administrative systems in countries other than Tanzania. Therefore, applying this mapping approach to other regions of Africa and beyond will require the adaptation to the particular systems of each country. In such cases, the smallest administrative units that exist in the respective areas of interest in those countries can be used as adequate substitutes for ten-cell-units. However, for successfully utilizing the participatory mapping procedure, it is of crucial importance that the residents of the target areas are aware of the administrative units they live in. Otherwise, community-based programme staff would not be able to draw from the knowledge of community members regarding the locations of boundaries. Therefore, in areas where the smallest existing administrative units are not well known to the local population, it might be a good alternative to refer to roads, rivers, pathways or similar intuitive landmarks that can be easily identified by community members.

Similar mapping approaches in African settings have been implemented for other purposes, scales and cities. For example, in Southern Sudan, urban maps have been produced to assist town planners in their efforts to respond effectively to returning population and reintegration issues. The Data Exchange Platform for the Horn of Africa (DEPHA) also provides a few datasets on urban scale. The EPIDEMIO programme has produced maps of several African cities. However, in contrast to the procedure in Dar es Salaam, these GIS-based approaches required a considerable amount of technical expertise and external support. Moreover, there are no participatory components. Hence, they cannot provide the necessary basis for community-based comprehensive mosquito larval surveillance.

The costs of the mapping approach are listed in table 2. For mapping the entire study area surveyed here, less than US$ 14,000 have been spent, which is equal to US$ 831 per km^2^. Thus, the complete set of correct sketch and formal maps covering one TCU costs an average of approximately US$ 24. Considering that the maps have to be produced only once and do not require much updating from then on, these costs appear reasonable and affordable not only for the Dar es Salaam programme, but also for any other comparable larval control intervention in Africa or elsewhere.

Areas that were initially not included in any sketch map are theoretically just as likely as any other area to contain breeding sites for malaria vectors, and might be very important sources for mosquitoes that fly into residential areas. In the study area in Dar es Salaam, most of the newly included areas were industrial or commercial areas and open spaces. Whereas industrial and commercial areas might be just as important as residential ones in terms of mosquito productivity, the open grass and scrublands that often frame such industrial and commercial plots are particularly likely to support key vectors from the *An. gambiae *complex [43]. This is particularly true for open spaces, notably those that are located in lowlands with a relatively high ground water table, and used for agricultural purposes. Considering that the number of infective mosquito bites per person per year is inversely proportional to the human population density [44,45], and mosquitoes disperse until they find blood [46,47], all these predominantly unoccupied areas might therefore contribute considerably to mosquito emergence rate [23,38,46-48] and malaria transmission [49] in neighbouring residential TCUs. Therefore, the inclusion of the initially unsurveyed areas into routine mosquito larval surveillance and control is likely to have a great impact on the effectiveness of such a programme, particularly after the planned addition of surrounding wards to the UMCP.

The framework generated through this mapping procedure made it possible to rationally allocate every square meter of the programme area to individual CORPs under the oversight of specific supervisors. Such individualization of responsibility is considered essential for managing larviciding programmes [50,51] because of the rigorous, sustained and comprehensive coverage required to achieve useful reductions of malaria transmission in Africa.

## Conclusion

The participatory mapping approach developed in Dar es Salaam enables complete coverage of targeted areas with mosquito larval habitat surveillance and control through comprehensive spatial coverage with community-derived maps. It can be fully integrated into an operational malaria control programme which takes local administrative or other suitable structures into account. The procedure is simple, straightforward, and low cost. It requires only minimal technical skills and equipment. Most importantly, even if the respective administrative boundary system varies from country to country, it can easily be scaled up not only to the remaining parts of Dar es Salaam, which is currently in progress, but also to other cities in Tanzania or any country affected by mosquito-borne diseases in Africa or elsewhere.

## Competing interests

The programme within which this study was conducted is partially supported by Valent Biosciences Corporation, a commercial manufacturer of microbial larvicides. Also, a substantial portion of the current salary and research support for the investigators depends on the achievement of documented suppression of malaria transmission and infection risk by this programme through systematic larviciding.

## Authors' contributions

SD designed and implemented the study, analyzed the results and drafted the manuscript. DN, KK, DM, HM, UF, AWD, MT and MCC participated in designing and implementing the study. GFK conceived the participatory mapping strategy, supported the design and implementation of the study, and assisted in drafting the manuscript. All authors read and approved the final manuscript.

## Supplementary Material

Additional file 1 **Guidelines for ten-cell-unit mapping**. "Guidelines for 10-cell unit mapping to be carried out by the Community-Owned Resource Persons and the wards malaria vector control supervisors" (pdf format) [31]. These guidelines describe the procedure for sketch mapping in detail, and have been distributed by the UMCP management to the CORPs and their supervising staff. Click here for file

## References

[B1] World Health Organization, UNICEF (2005). World Malaria Report 2005.

[B2] Hay SI, Guerra CA, Tatem AJ, Atkinson PM, Snow RW (2005). Urbanization, malaria transmission and disease burden in Africa. Nat Rev Microbiol.

[B3] Snow RW, Guerra CA, Noor AM, Myint HY, Hay SI (2005). The global distribution of clinical episodes of Plasmodium falciparum malaria. Nature.

[B4] Lines J, Harpham T, Leake C, Schofield C (1994). Trends, priorities and policy directions in the control of vector-borne diseases in urban environments. Health Policy Plan.

[B5] Warren M, Billig P, Bendahmane D, Wijeyaratne P (1999). Malaria in Urban and Peri-Urban areas in Sub Saharan Africa. Environmental Health Project Activity.

[B6] Robert V, Macintyre K, Keating J, Trape JF, Duchemin JB, Warren M, Beier JC (2003). Malaria transmission in urban sub-Saharan Africa. Am J Trop Med Hyg.

[B7] Granja AC, Machungo F, Gomes A, Bergstrom S, Brabin B (1998). Malaria-related maternal mortality in urban Mozambique. Ann Trop Med Parasitol.

[B8] Imbert P, Sartelet I, Rogier C, Ka S, Baujat G, Candito D (1997). Severe malaria among children in a low seasonal transmission area, Dakar, Senegal: influence of age on clinical presentation. Trans R Soc Trop Med Hyg.

[B9] Trape JF, Lefebvre-Zante E, Legros F, Druilhe P, Rogier C, Bouganali H, Salem G (1993). Malaria morbidity among children exposed to low seasonal transmission in Dakar, Senegal and its implications for malaria control in tropical Africa. Am J Trop Med Hyg.

[B10] Mtasiwa D, Siegfried G, Tanner M, Pichette P (2003). The Dar es Salaam City/Region minimum package of health and related management activities: From managing diseases to managing health systems.

[B11] Makani J, Matuja W, Liyombo E, Snow RW, Marsh K, Warrell DA (2003). Admission diagnosis of cerebral malaria in adults in an endemic area of Tanzania: implications and clinical description. Qjm.

[B12] Reyburn H, Mbakilwa H, Mwangi R, Mwerinde O, Olomi R, Drakeley C, Whitty CJ (2007). Rapid diagnostic tests compared with malaria microscopy for guiding outpatient treatment of febrile illness in Tanzania: randomised trial. BMJ.

[B13] Wang SJ, Lengeler C, Mtasiwa D, Mshana T, Manane L, Maro G, Tanner M (2006). Rapid urban malaria appraisal (RUMA) II: Epidemiology of urban malaria in Dar es Salaam (Tanzania). Malar J.

[B14] Castro MC, Yamagata Y, Mtasiwa D, Tanner M, Utzinger J, Keiser J, Singer BH (2004). Integrated urban malaria control: a case study in Dar es Salaam, Tanzania. Am J Trop Med Hyg.

[B15] Clyde DF (1961). Malaria control in Tanganyika under the German Administration. Part I.. East Afr Med J.

[B16] Mukabana WR, Kannady K, Kiama GM, Ijumba JN, Mathenge EM, Kiche I, Nkwengulila G, Mboera L, Mtasiwa D, Yamagata Y, van Schayk I, Knols BG, Lindsay SW, Caldas de Castro M, Mshinda H, Tanner M, Fillinger U, Killeen GF (2006). Ecologists can enable communities to implement malaria vector control in Africa. Malar J.

[B17] Kilama WL, Kihamia CM, Mwaluko GMP, Kilama WL, Mandara MP, Murru M, Macpherson CNL (1991). Malaria. Health and disease in Tanzania.

[B18] Tanzanian Ministry of Health, World Health Organization (2004). National Malaria Medium Term Strategic Plan, July 2002 - June 2006.

[B19] Mtasiwa D, Kiama GM, Killeen GF (2004). Integrated malaria control in Dar es Salaam: Programme Guidebook.

[B20] Vanek MJ, Shoo B, Mtasiwa D, Kiama M, Lindsay SW, Fillinger U, Kannady K, Tanner M, Killeen GF (2006). Community-based surveillance of malaria vector larval habitats: a baseline study in urban Dar es Salaam, Tanzania. BMC Public Health.

[B21] Gu W, Novak RJ (2005). Habitat-based modeling of impacts of mosquito larval interventions on entomological inoculation rates, incidence, and prevalence of malaria. Am J Trop Med Hyg.

[B22] Diuk-Wasser MA, Bagayoko M, Sogoba N, Dolo G, Touré MB, Traoré SF, Taylor CE (2004). Mapping rice field anopheline breeding habitats in Mali, West Africa, using Landsat ETM+ sensor data. International Journal of Remote Sensing.

[B23] Keating J, Macintyre K, Mbogo CM, Githure JI, Beier JC (2004). Characterization of potential larval habitats for Anopheles mosquitoes in relation to urban land-use in Malindi, Kenya. Int J Health Geogr.

[B24] Mushinzimana E, Munga S, Minakawa N, Li L, Feng CC, Bian L, Kitron U, Schmidt C, Beck L, Zhou G, Githeko AK, Yan G (2006). Landscape determinants and remote sensing of anopheline mosquito larval habitats in the western Kenya highlands. Malar J.

[B25] Sattler MA, Mtasiwa D, Kiama M, Premji Z, Tanner M, Killeen GF, Lengeler C (2005). Habitat characterization and spatial distribution of Anopheles sp. mosquito larvae in Dar es Salaam (Tanzania) during an extended dry period. Malar J.

[B26] Sithiprasasna R, Lee WJ, Ugsang DM, Linthicum KJ (2005). Identification and characterization of larval and adult anopheline mosquito habitats in the Republic of Korea: potential use of remotely sensed data to estimate mosquito distributions. Int J Health Geogr.

[B27] Killeen GF, Tanner M, Mukabana WR, Kalongolela MS, Kannady K, Lindsay SW, Fillinger U, Castro MC (2006). Habitat targeting for controlling aquatic stages of malaria vectors in Africa. Am J Trop Med Hyg.

[B28] United Nations (2005). World Urbanization Prospects: The 2005 Revision.

[B29] MARA/ARMA (2002). MARA LITe for Africa.

[B30] National Bureau of Statistics (2003). The 2002 population and housing census general report.

[B31] Urban Malaria Control Project (2004). Guidelines for 10-cell unit mapping to be carried out by the community-owned resource persons and the wards malaria vector control supervisors.

[B32] McCloy KR (2006). Resource Management Information Systems: Remote Sensing, GIS and Modelling.

[B33] Lillesand TM, Kiefer RW, Chipman JW (2004). Remote Sensing and Image Interpretation.

[B34] Booman M, Durrheim DN, La Grange K, Martin C, Mabuza AM, Zitha A, Mbokazi FM, Fraser C, Sharp BL (2000). Using a geographical information system to plan a malaria control programme in South Africa. Bull World Health Organ.

[B35] Booman M, Sharp BL, Martin CL, Manjate B, La Grange JJ, Durrheim DN (2003). Enhancing malaria control using a computerised management system in southern Africa. Malar J.

[B36] Martin C, Curtis B, Fraser C, Sharp B (2002). The use of a GIS-based malaria information system for malaria research and control in South Africa. Health Place.

[B37] Tanser FC, Le Sueur D (2002). The application of geographical information systems to important public health problems in Africa. Int J Health Geogr.

[B38] Eisele TP, Keating J, Swalm C, Mbogo CM, Githeko AK, Regens JL, Githure JI, Andrews L, Beier JC (2003). Linking field-based ecological data with remotely sensed data using a geographic information system in two malaria endemic urban areas of Kenya. Malar J.

[B39] Keating J, MacIntyre K, Mbogo C, Githeko A, Regens JL, Swalm C, Ndenga B, Steinberg LJ, Kibe L, Githure JI, Beier JC (2003). A geographic sampling strategy for studying relationships between human activity and malaria vectors in urban Africa. Am J Trop Med Hyg.

[B40] Abbot J, Chambers R, Dunn C, Harris T, de Merode E, Porter G, Townsend J, Weiner D (1998). Participatory GIS: opportunity or oxymoron?. PLA Notes.

[B41] Dunn CE, Atkins PJ, Blakemore MJ, Townsend JG (1999). Teaching Geographical Information Handling Skills for Lower-income Countries. Transactions in GIS.

[B42] Ifakara Health Research and Development Centre (2005). The Community Voice - Getting Community Needs into District Development Plans. An Operational Manual for District Management Teams.

[B43] Briet OJ, Dossou-Yovo J, Akodo E, van de Giesen N, Teuscher TM (2003). The relationship between Anopheles gambiae density and rice cultivation in the savannah zone and forest zone of Cote d'Ivoire. Trop Med Int Health.

[B44] Killeen GF, McKenzie FE, Foy BD, Schieffelin C, Billingsley PF, Beier JC (2000). A simplified model for predicting malaria entomologic inoculation rates based on entomologic and parasitologic parameters relevant to control. Am J Trop Med Hyg.

[B45] Smith DL, McKenzie FE (2004). Statics and dynamics of malaria infection in Anopheles mosquitoes. Malar J.

[B46] Service MW (1997). Mosquito (Diptera: Culicidae) Dispersal: The Long and Short of it. Journal of Medical Entomology.

[B47] Smith DL, Dushoff J, McKenzie FE (2004). The risk of a mosquito-borne infection in a heterogeneous environment. PLoS Biol.

[B48] Killeen GF, Knols BG, Gu W (2003). Taking malaria transmission out of the bottle: implications of mosquito dispersal for vector-control interventions. Lancet Infect Dis.

[B49] Le Menach A, McKenzie FE, Flahault A, Smith DL (2005). The unexpected importance of mosquito oviposition behaviour for malaria: non-productive larval habitats can be sources for malaria transmission. Malar J.

[B50] Killeen GF, Fillinger U, Kiche I, Gouagna LC, Knols BGJ (2002). Eradication of Anopheles gambiae from Brazil: lessons for malaria control in Africa?. The Lancet Infectious Diseases.

[B51] Soper FL, Wilson DB (1943). Anopheles gambiae in Brazil: 1930 to 1940.

